# Molecular characterization of mutations in galactosemia genes: structural and functional implications

**DOI:** 10.1186/1755-8166-7-S1-O8

**Published:** 2014-01-21

**Authors:** R Singh, BR Thapa, G Kaur, R Prasad

**Affiliations:** 1 Department of Biochemistry, Postgraduate Institute of Medical Education and Research, Chandigarh, India; 2 Department of Gastroenterology, Postgraduate Institute of Medical Education and Research, Chandigarh, India; 3 Department of Physiology, GMCH, Chandigarh, India

## Background

Galactosemia is an autosomal recessive metabolic disorder caused by the deficiency of enzymes involved in galactose metabolism resulting in complications like cataracts, hepatocellular damage and developmental delay. Nonetheless, no report is available on mutations in galactosemia genes from our population. The objective of the present study was to determine blood GALT and GALK activity in infants with cholestasis and congenital cataracts and to establish a spectrum of mutations in GALT and GALK genes.

## Material and methods

430 infants (2 days-11 months) with cholestasis admitted in Pediatric Gastroenterology over 3.5 years were evaluated for galactosemia. Basic investigations included hemogram, liver function tests, blood culture, urine culture, urine for non-glucose reducing substances and eye evaluation. Screening for GALT deficiency was done using Perkin–Elmer neonatal GALT kit. The levels of galactose-1-phosphate were also measured. Also, 115 patients with congenital cataracts were screened for the galactokinase (GALK) deficiency. Mutation analysis for most common Q188R and N314D mutations in GALT gene was performed by Restriction Fragment Length Polymorphism (RFLP). Single Stranded Conformational Polymorphism (SSCP) analysis and subsequently DNA sequencing were done for identification and characterization of unknown and novel mutations in GALT and GALK genes.

## Results

55 (12.7%) infants were found to have reduced GALT activity with male: female: 37:18, jaundice in 55 (100%), hepatomegaly in 54 (98%), splenomegaly in 32 (58%), coagulopathy in 23 (42%), encephalopathy in 9 (16%), septicemia in 10 (18%) and cataracts in 12 (22%) were observed. Increased galactose-1 phosphate levels were fraternized with reduced activity of GALT. A total of 16 mutations and 4 polymorphisms were detected. 10 were novel mutations. Reduced blood galactokinase activity was found in 13 (10.2%) patients with congenital cataracts. 4 novel mutations were found in GALK gene.

## Conclusions

N314D mutation was found to be the most common mutation in our population. 10 and 4 novel mutations were also detected in GALT and GALK genes respectively.

